# Microstructure and Mechanical Properties of NiTi-Based Eutectic Shape Memory Alloy Produced via Selective Laser Melting In-Situ Alloying by Nb

**DOI:** 10.3390/ma14102696

**Published:** 2021-05-20

**Authors:** Igor Polozov, Anatoly Popovich

**Affiliations:** Institute of Mechanical Engineering, Materials, and Transport, Peter the Great St. Petersburg Polytechnic University, Polytechnicheskaya 29, 195251 St. Petersburg, Russia; director@immet.spbstu.ru

**Keywords:** additive manufacturing, powder bed fusion, nitinol

## Abstract

This paper presents the results of selective laser melting (SLM) process of a nitinol-based NiTiNb shape memory alloy. The eutectic alloy Ni_45_Ti_45_Nb_10_ with a shape memory effect was obtained by SLM in-situ alloying using a powder mixture of NiTi and Nb powder particles. Samples with a high relative density (>99%) were obtained using optimized process parameters. Microstructure, phase composition, tensile properties, as well as martensitic phase transformations temperatures of the produced alloy were investigated in as-fabricated and heat-treated conditions. The NiTiNb alloy fabricated using the SLM in-situ alloying featured the microstructure consisting of the NiTi matrix, fine NiTi+β-Nb eutectics, as well as residual unmelted Nb particles. The mechanical tests showed that the obtained alloy has a yield strength up to 436 MPa and the tensile strength up to 706 MPa. At the same time, in-situ alloying with Nb allowed increasing the hysteresis of martensitic transformation as compared to the alloy without Nb addition from 22 to 50 °C with an increase in A_f_ temperature from −5 to 22 °C.

## 1. Introduction

NiTi nitinol-based alloys are among the most important shape memory and superplastic materials and are of considerable interest for potential applications in aerospace and biomedical fields [1,2]. Nitinol has found its practical applications in the aircraft industry for thermo-power actuators, thermomechanical connectors, in medicine for stents, constrictive fixators, and other medical devices [3,4]. Nitinol-based alloys, unlike Fe- and Cu-based shape memory alloys, are characterized by high strength and ductility and have high corrosion resistance and biocompatibility. However, brittle secondary phases such as the metastable compound Ni_4_Ti_3_, which can decompose into Ni_3_Ti_2_ and Ni_3_Ti with increasing temperature, are commonly observed in NiTi-based alloys [5,6]. These phases increase the hardness of the material, but reduce the ductility of NiTi alloys, limiting their application. In this regard, it is relevant to introduce secondary phases with high hardness into the matrix of NiTi-based alloys while maintaining or slightly reducing the level of ductility.

It is known that Nb plays an important role in nitinol-based alloys because the addition of Nb increases the hysteresis of martensitic transformation [7]. Besides, the addition of Nb improves the biocompatibility of NiTi-based alloys [8]. Extension of the temperature interval of martensitic transformation can be used in the manufacture of connecting elements or seals [9]. Ni_47_Ti_44_Nb_9_ alloy is a classic nitinol-based shape memory alloy characterized by an in situ composite microstructure [7]. It is known that the microstructure of the alloy with shape memory effect Ni_47_Ti_44_Nb_9_ consists of NiTi matrix and β-Nb phase [10]. The presence of β-Nb is known to have a significant effect on the shape memory effect and mechanical properties of the alloy [11]. Nb exhibits different properties from the NiTi matrix with a shape memory effect [12]. The presence of Nb as a second phase (β-Nb) or as a solution is the primary reason for transformation hysteresis expansion in NiTiNb shape memory alloys [10].

Conventionally, Ni_47_Ti_44_Nb_9_ alloy parts are made by casting or sintering powder materials, which have limitations in terms of part geometry, as well as control of their microstructure and properties [13,14,15]. In this regard, it is promising to use additive manufacturing (AM) techniques, in particular, the selective laser melting (SLM) method for the manufacture of parts from alloys with the shape memory effect [16].

The SLM process uses metallic powders as a feedstock material. Various research works have shown that pre-alloyed powders allow obtaining a material with a more homogeneous microstructure and stable mechanical properties [17,18,19]. However, the production of pre-alloyed powders is usually time and labor consuming, especially in the case of custom alloys. The use of an elemental powder blend in the SLM process is an alternative option that can be used for in-situ synthesis or in-situ alloying during the SLM process to obtain a material with a required composition [20,21,22]. At the same time, rapid solidification and cooling rates typical for the SLM process lead to insufficient diffusion of elements with high melting points, which causes a heterogeneous microstructure. In this regard, additional heat treatment can be applied to improve the chemical homogeneity of the material [23,24]. AM methods, such as SLM or Direct Energy Deposition (DED), involve repetitive heating and high solidification rates leading to distinctive microstructural features of nitinol alloys [25]. For example, rapid solidification promotes the formation of a supersaturated solid solution matrix [25]. At the same time, various process parameters can result in different microstructures and phase composition of nitinol alloys [26], as well as different Ni content resulting in significant changes in transformation behavior [16].

In this work, the feasibility of the SLM process to produce a nitinol-based shape memory alloy via in-situ alloying by Nb was investigated. Using a powder blend of NiTi-alloy and pure Nb, the influence of the SLM process parameters on the sample’s density was studied. Microstructure, phase composition, mechanical properties, and martensitic phase transformation temperatures were studied for the NiTiNb alloy in the as-fabricated and heat-treated conditions.

## 2. Materials and Methods

The following materials were used as feedstock materials: gas atomized NiTi alloy powder with a nickel content of 51.4% (at.) and niobium powder with a purity of 99.8%. The NiTi powder had the following particle size distribution: d_10_ = 27.2 µm, d_50_ = 50.0 µm, and d_90_ = 84.9 µm. The niobium powder in the initial state had a spherical particle shape and was pretreated in a thermal plasma jet using Tekna Tek-15 (Sherbrooke, QC, Canada) plasma spheroidization unit to obtain spherical particles. The details of the plasma spheroidization process can be found in [27]. The final niobium powder had the following particle size distribution: d_10_ = 15.5 µm, d_50_ = 35.5 µm, and d_90_ = 72.8 µm. Particle size distribution of the powders was measured by laser diffraction technique with Analysette 22 NanoTec (Fritsch, Idar-Oberstein, Germany).

**Figure 1:**
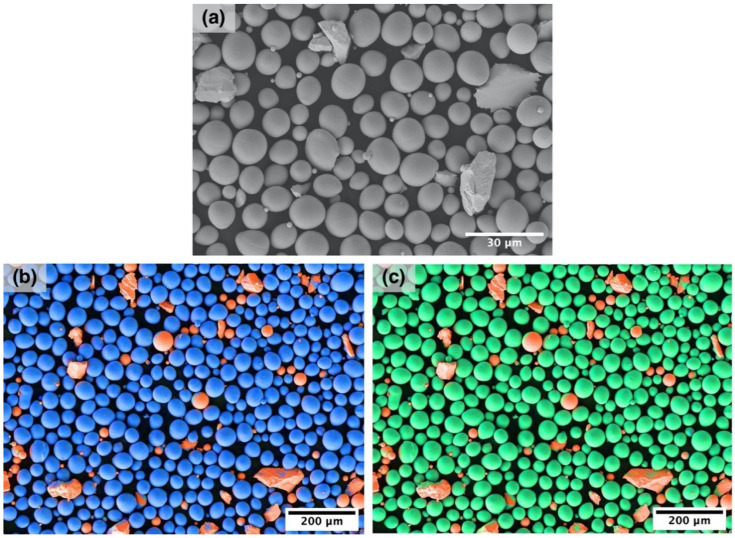
(**a**) SEM-image of the NiTi-Nb powder blend and chemical distribution of the elements in the blend: (**b**) blue—Ti and red—Nb and (**c**) green—Ni and red—Nb.

NiTi and Nb powders were mixed in 85% NiTi: 15% Nb weight ratio for 12 h using a tumbler mixer to obtain a powder blend with Ni_47_Ti_44_Nb_9_ composition. Figure 1a shows a scanning electron microscope (SEM) image of the NiTi and Nb powder blend. The NiTi powder particles have a spherical shape, while some Nb particles have irregular shape due to incomplete spheroidization. Figure 1b,c shows the distribution of Ti, Ni, and Nb elements in the powder blend demonstrating that Nb particles were fairly distributed among the NiTi powder.

The NiTiNb alloy samples were manufactured from the prepared powder blend using AconityMIDI (Aconity3D GmbH, Herzogenrath, Germany) SLM system with varying process parameters. Samples with the size of 10 × 10 × 10 mm^3^ were produced to study the microstructure, phase composition, and density of the material. Two groups of samples were fabricated, the difference between which is the use of different powder layer thickness and the laser beam spot size. Laser power, scanning speed, and hatch distance were also varied. The SLM process parameters used in the study are shown in Table 1. Sets A, B, and C use standard laser spot size (~70 μm) and sets G, K, and J use increased laser spot size with an unfocused beam (~300 μm). The volume energy density (VED) calculated according to a standard equation [28,29] was used as a parameter to investigate the effects of SLM process parameters on the samples’ density.

To study the effect of heat treatment temperature and holding time on the microstructure and properties of the samples, annealing was carried out under the following conditions:

- heating to 500 °C and holding at 500 °C for 2 h;
- heating to 900 °C and holding at 900 °C for 30 min;
- heating to 900 °C and holding at 900 °C for 2 h.

The samples were heated at a rate of 10 °C/min and furnace cooled. The annealing temperature of 900 °C is above the recrystallization temperature of NiTiNb alloys [30], while 500 °C is below the recrystallization temperature and its main purpose is residual stress relieving.

The microstructure was studied using a TESCAN Mira 3 LMU scanning electron microscope (SEM) (TESCAN, Brno, Czech Republic) and a Leica DMI 5000 optical microscope (Leica, Wetzlar, Germany). To study the microstructure, polished microsections of the samples were etched using the following etchant: 83% H_2_O, 14% HNO3, and 3% HF. The samples were cut along the building direction. The chemical composition of the material was investigated using energy-dispersive analysis (EDS) (TESCAN, Brno, Czech Republic). The phase composition of the material was determined using a Bruker D8 Advance diffractometer on CuKα radiation (λ = 1.5418 Å). The density of the material was measured by the Archimedes principle. Tensile mechanical properties were investigated using cylindrical specimens using a Zwick/Roell Z050 testing machine (Ulm, Germany). Temperatures of martensitic transformations of the fabricated alloy were determined by differential scanning calorimetry (DSC). To compare phase transformation temperatures, NiTi samples were fabricated without the addition of Nb particles using the E2 parameter set.

## 3. Results and Discussion

**Figure 2:**
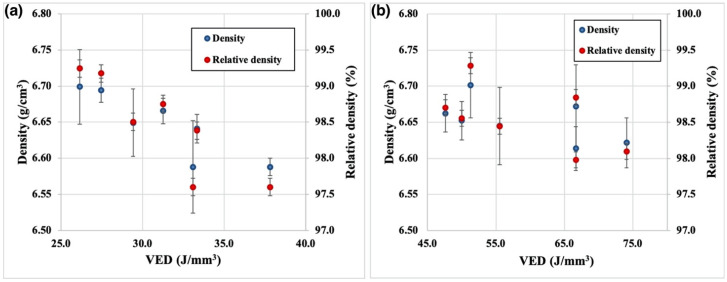
Effect of VED on the density of NiTiNb samples produced by SLM using (**a**) 300 µm and (**b**) 70 µm laser spot size.

Figure 2 shows the effect of VED on the density of NiTiNb samples produced using the NiTi-Nb powder blend with 300 µm (Figure 2a) and 70 µm (Figure 2b) laser spot size. The highest relative density values of around 99.2 ± 0.05% were obtained using E1 and E2 parameter sets, which correspond to 300 µm laser spot size and 26–27 J/mm^3^ VED. Increasing VED led to lower density values, which might be attributed to melt pool overheating and instability and formation of gas and keyhole pores [31,32]. At the same time, varying VED at 70 µm laser spot size and 50 µm layer thickness did not lead to a significant change in density.

**Figure 3:**
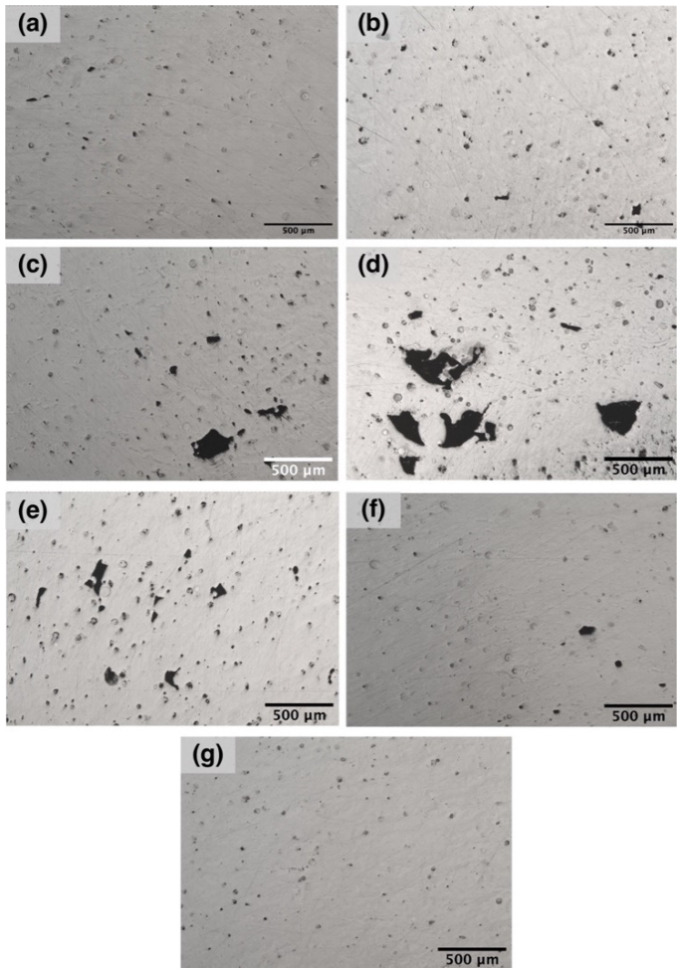
Optical images of the polished microsections for the samples produced using parameter sets (**a**) D1, (**b**) D2, (**c**) D3, (**d**) F1, (**e**) F2, (**f**) E1, and (**g**) E2.

As can be seen in Figure 3, there are several types of internal defects in samples produced under different process parameters. In the case of the samples D3 and F1, coarse pores with the size of 300–400 µm and irregular shape can be found along with fine spherical pores. These coarse pores elongated in the direction parallel to the laser path might be the result of lack-of-fusion due to the interaction of various factors: capillary forces, material evaporation, insufficient melting, etc. [33,34]. The smallest number of defects can be seen in the case of the sample E2, which corresponds to 27.5 J/mm^3^ VED and 300 µm laser spot and has the highest density value.

**Figure 4:**
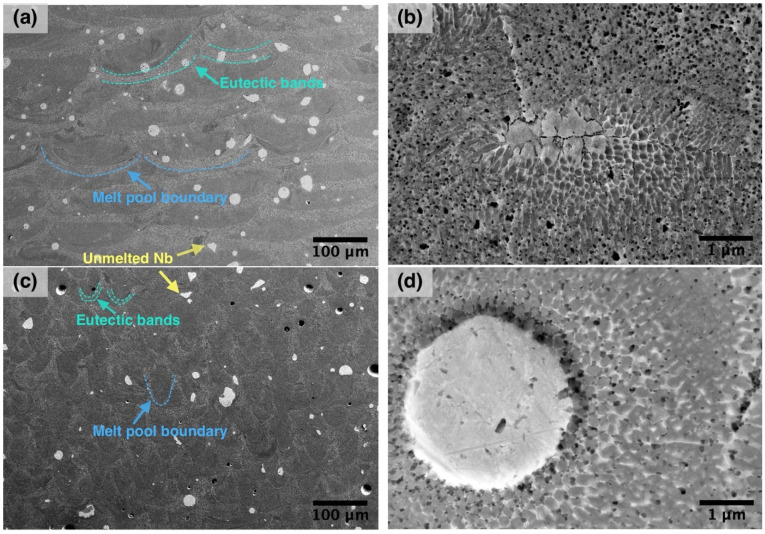
SEM-images showing the microstructure of the NiTiNb samples produced using parameter sets (**a**,**b**) E1 and (**c**,**d**) A1.

Figure 4 shows SEM-images of microstructures of the samples manufactured using E1 and A1 parameter sets. In the case of the E1 sample produced with an unfocused laser beam and 100 µm layer thickness, there are coarse solidified melt pools with a width comparable to the diameter of the laser spot as can be seen in Figure 4a. Melt pool boundaries can be clearly distinguished due to the presence of eutectic bands.

**Figure 5:**
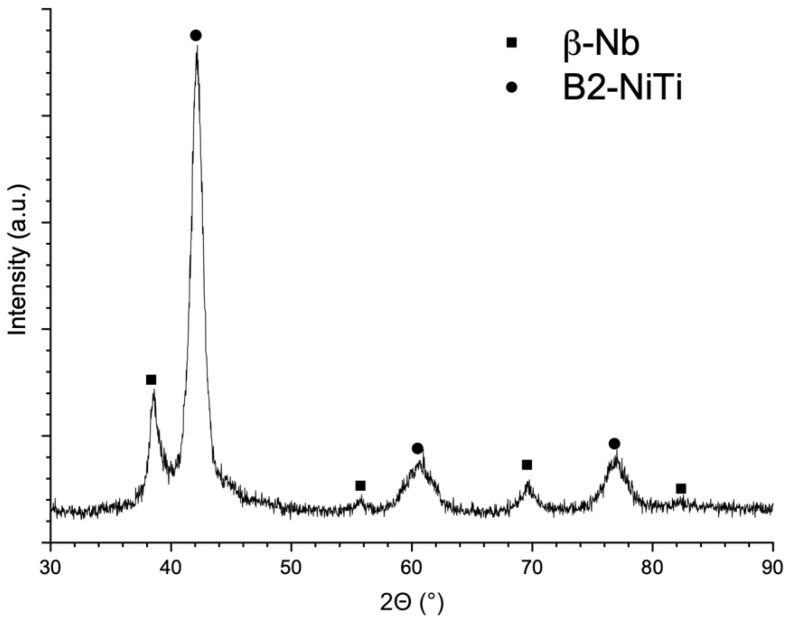
The XRD pattern of the NiTiNb sample produced using the E1 parameter set.

TiNi-Nb alloy system is a eutectic alloy and according to the phase diagram, the eutectic point corresponds to 26 at. % Ni content [35]. The eutectic temperature is 1150.7 °C. Hence, NiTi+β-Nb eutectic should be present in the microstructure after solidification and cooling of the Ti-Ni-Nb alloy [36]. The presence of NiTi and β-Nb phase was confirmed by the XRD results (Figure 5). The eutectic is mainly located at the melt pool boundaries as can be seen in Figure 4. Nb has a significantly higher density and melting point compared to NiTi. As a consequence, during the SLM of the powder blend, melting of NiTi will occur first, while the Nb may remain unmelted. Mixing of the alloy components in the melt can be carried out by Marangoni convection [37], which would result in a distribution of Nb in the volume of a melt pool. During the laser melting of the powder layer, the underlying solidified material is partially remelted. This can promote the diffusion at the melt pool boundaries and the dissolution of the elements in these micro volumes. Thus, the eutectic NiTI+β-Nb microstructure can be mainly found at the melt pool boundaries. As can be seen in Figure 4b,d, NiTi+β-eutectic areas are visible near partially melted on unmelted Nb particles where diffusion takes place during the SLM process and promotes the formation of fine eutectic microstructure.

When 50 µm layer thickness and a standard laser spot size were applied during the SLM process (sample A1), the microstructure of the obtained alloy also features melt pool boundaries as can be seen in Figure 4c, but their size is much smaller compared to E1 sample. The eutectic bands width is also smaller in this case, which indicates less degree of Nb diffusion into the NiTi matrix in case of smaller laser spot size. When an unfocused laser beam along with increased laser power and layer thickness is used, the melt pool volume is bigger and a higher volume of powder blend is melted with the laser. At the same time, a bigger melt pool volume leads to a lower cooling rate during the SLM process [38], which promotes the diffusion of elements and formation of the eutectic phase. Thus, a higher volume of eutectic phase is obtained when an increased laser spot size is used.

**Figure 6:**
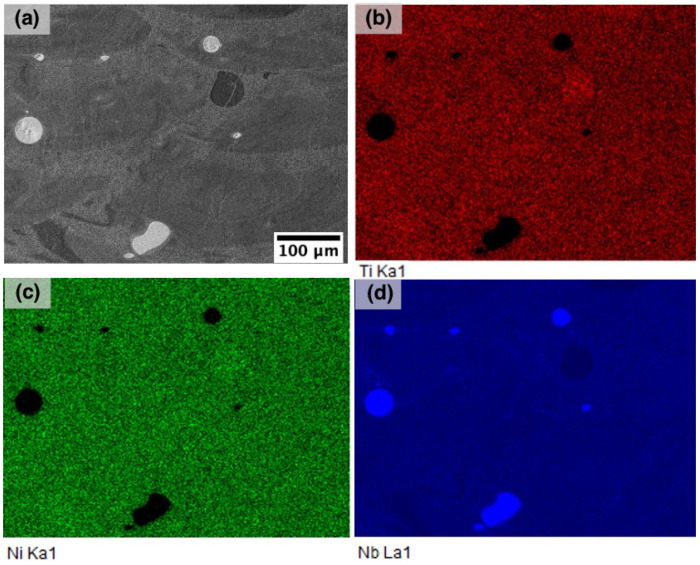
(**a**) SEM-image of the microstructure of E1 sample and EDS-maps showing the distribution of (**b**) Ti, (**c**) Ni, and (**d**) Nb.

Figure 6 shows the chemical distribution of the elements in the NiTiNb sample produced using the E1 parameter set. It can be seen that there are areas of pure Nb corresponding to unmelted Nb particles. In general, Ti, Ni, and Nb are homogeneously distributed in the volume, and the eutectic regions do not feature a significant change in concentration of the elements compared to the rest of the sample. According to the EDS results, the obtain alloy has the following composition (in at. %): 45.5 ± 0.2 Ti, 44.9 ± 0.2 Ni, and 9.6 ± 0.4 Nb. The obtained composition is characterized by a decreased Ni content due to its evaporation during the SLM process, which is consistent with the results on the SLM of NiTi alloys reported in the literature [16,39].

**Figure 7:**
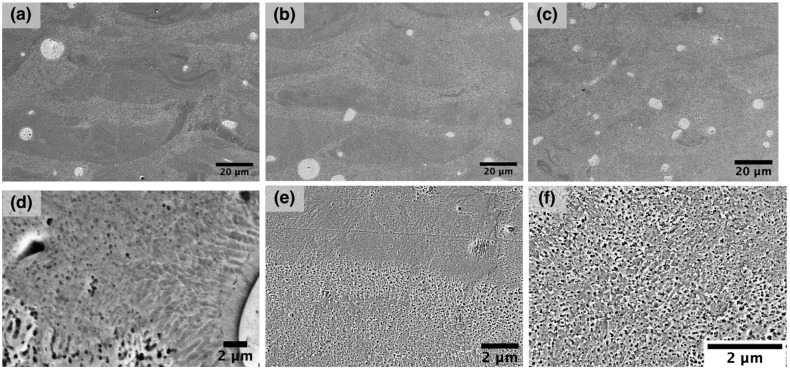
SEM-images of the NiTiNb samples after different heat treatments: (**a**,**d**) 500 °C for 2 h, (**b**,**e**) 900 °C for 30 min, and (**c**,**f**) 900 °C for 2 h.

To obtain a more homogeneous structure, the samples manufactured using the E1 parameter set were heat-treated under different conditions. SEM-images of the microstructures after heat treatment are shown in Figure 7. As a result of heat treatment, Nb diffusion into the material matrix occurs, which is observed in the form of partial dissolution of Nb particles and an increase in the eutectic volume fraction, but the annealing temperature and time are not sufficient for a complete dissolution of Nb, because it has a low diffusion coefficient. Thus, after annealing at 900 °C for 30 min, the alloy structure has not undergone significant changes. As the annealing time was increased to 2 h at 900 °C, the former melt pool boundaries became less pronounced, and the fraction of regions with eutectic microstructure increased due to an accelerated niobium diffusion in the alloy matrix.

For the tensile tests, the samples were heat-treated by annealing at 900 °C for 2 h since it provides a more homogeneous microstructure of the alloy. The results of the tensile tests are shown in Table 2. While the tensile strength of the in-situ alloyed samples is comparable to the casted Ni_47_Ti_44_Nb_9_ alloy, the elongation is significantly lower. Heterogeneous microstructure and impurities pick-up are believed to be the main factors affecting the elongation of the SLM-ed alloy.

**Figure 8:**
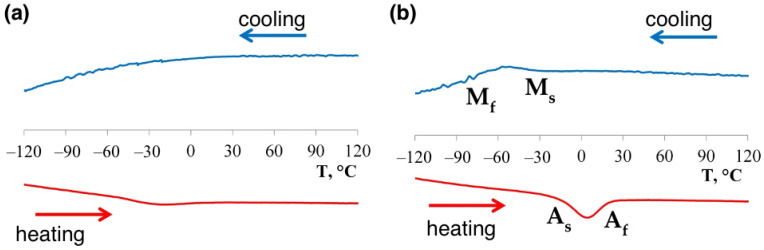
DSC-curves of the in-situ alloyed NiTiNb samples (**a**) in the as-fabricated state and (**b**) after annealing at 900 °C for 2 h.

Figure 8 shows the DSC curves for the fabricated samples. In the as-fabricated condition, the reverse martensitic transformation effect is poorly visible. However, the presence of a reverse martensitic transformation peak allows for the conclusion that a forward martensitic transformation also takes place during cooling. Annealing at 900 °C resulted in A_f_ temperature shift into higher temperature range and made both forward and reverse transformation effects more pronounced. Table 3 summarizes the DSC results of the obtained samples showing martensitic phase transformation temperatures in the as-fabricated and heat-treated states. One of the main functional properties of shape memory alloys are temperatures of direct and reverse martensitic phase transformations. It is reasonable to consider that the solution of Nb in the NiTi matrix can change the kinetics of martensitic transformations [10].

Annealing at 900 °C leads to increased and narrowed temperature intervals of the reverse martensitic transformation compared to the as-fabricated state. The A_f_ temperature shifts to the region of positive temperatures. At the same time, “in-situ” alloying increased the hysteresis of martensitic transformation from 22 to 50 °C as compared to NiTi alloy without Nb addition with an increase in the A_f_ temperature from −5 to 22 °C. The in-situ alloyed NiTiNb samples showed higher direct martensitic transformation temperatures compared to the casted Ni_47_Ti_44_Nb_9_ alloy, which might be attributed to the difference in Ni content. Due to partial evaporation, the SLM-ed samples have a Ni content of around 45.4 at. % leading to the increase of transformation temperatures.

## 4. Conclusions

The SLM process of NiTi-based eutectic alloy with shape memory effect obtained by in-situ alloying with Nb has been studied. Based on the results obtained, the following main conclusions can be made:

- NiTiNb shape memory alloy can be produced by SLM in-situ alloying by Nb with a relative density of 99%.
- The microstructure of the in-situ alloyed material consists of B2-NiTi matrix, fine NiTi + β-Nb eutectic phase, and residual unmelted Nb particles.
- The use of increased laser spot size with simultaneous increase of layer thickness and laser power allows to obtain more homogeneous element distribution and homogeneous microstructure of NiTiNb alloy.
- The SLM in-situ alloying of NiTi by Nb allowed increasing the martensitic transformation hysteresis as compared to the alloy without Nb addition from 22 to 50 °C while the A_f_ temperature increased from −5 to 22 °C.
- Annealing of the in-situ alloyed material at 900 °C resulted in improved microstructural homogeneity and higher tensile strength.

## Figures and Tables

**Table 1:** SLM process parameters used to fabricate the samples.

Process Parameter Set	Power (W)	Scanning Speed (mm/s)	Hatch Distance (µm)	Layer Thickness (µm)	VED (J/mm^3^)
A1	200	600	120	50	55.6
A2	200	650	120		51.3
A3	200	700	120		47.6
B1	180	600	120		50.0
B2	240	600	120		66.7
C1	200	600	100		66.7
C2	200	600	90		74.1
D1	450	300	450	100	33.3
D2	450	320	450		31.3
D3	450	340	450		29.4
E1	400	340	450		26.1
E2	420	340	450		27.5
F1	450	340	400		33.1
F2	450	340	350		37.8

**Table 2:** The results of tensile tests at room temperature for NiTiNb samples in the as-fabricated and heat-treated state.

Material	Yield Strength (MPa)	Tensile Strength (MPa)	Elongation (%)
NiTi-+Nb, SLM, as-fabricated	390 ± 10	590 ± 60	1.5 ± 0.1
NiTi-+Nb, SLM, after H/T (900 °C for 2 h)	410 ± 20	680 ± 20	3.8 ± 0.3
Ni_47_Ti_44_Nb_9_, casted [10]	~500	~650	~40

**Table 3:** Martensitic phase transformation temperatures of the fabricated alloys.

Sample	M_s_ (°C)	M_f_ (°C)	A_s_, (°C)	A_f_ (°C)	А_f_-A_s_ (°C)
NiTi+Nb, SLM as-fabricated	−50	−78	−52	5	57
NiTi+Nb, SLM+H/T (900 °C for 2 h)	−30	−76	−28	22	50
NiTi, SLM as-fabricated	−34	−69	−27	−5	22
Ni_47_Ti_44_Nb_9_, casted [40]	−73	−90	−25	−11	14

## Data Availability

The data presented in this study are available on request from the corresponding author.
